# Phyto mediated synthesis of copper oxide nanoparticles with *Zingiber officinale*: comprehensive bioactivity assessment against bacterial pathogens, cancer, and viruses

**DOI:** 10.1186/s12896-026-01124-2

**Published:** 2026-03-21

**Authors:** Mohamed K. Y. Soliman, Mai M. El-Ashmony, Hager A. Bendary, Fatma Rasslan, Salem S. Salem

**Affiliations:** 1 Botany and Microbiology Department, Faculty of Science, Al-Azhar University, Nasr City, Cairo, 11884 Egypt; 2 Department of Microbiology and Immunology, Faculty of pharmacy (Girls), Al- Azhar University, Cairo, 11765 Egypt

**Keywords:** CuO NPs, Characterization, Antibacterial, Anticancer, Antiviral, Antidiabetic

## Abstract

This work investigated the capacity of *Zingiber officinale* extract to synthesize copper oxide nanoparticles. *Z. officinale* extract is rich in diverse phytochemical constituents, as confirmed by GC–MS analysis. The synthesized CuO NPs were characterized using TEM, FTIR, XRD, SEM, and EDX analyses. CuO NPs exhibited antibacterial efficacy against various bacterial strains. The DPPH methodology was employed to evaluate the free radical scavenging capacity, yielding an IC₅₀ value of 244.5 µg/mL. The CuO NPs exhibited pronounced antibiofilm activity at 100 µg/mL, reducing biofilm formation by *S. aureus* (MRSA) and *E. coli* by 70.93% and 67.3%, respectively. In addition, they demonstrated substantial antidiabetic potential, inhibiting α-amylase and α-glucosidase by up to 90% and 84% at 1000 µg/mL, with corresponding IC₅₀ values of 46.42 µg/mL and 38.9 µg/mL, respectively. Furthermore, CuO NPs exhibited low toxicity toward Vero cells, with an IC₅₀ of 261 µg/mL and showed potential anticancer activity against the carcinoma cell lines CaCo2 and McF7, with IC₅₀ values of 84.4 and 168 µg/mL, respectively. At 125 µg/mL, CuO NPs inhibited HAV and HSV-1 by 37% and 41.1%, respectively. These multifaceted bioactivities highlight the potential of *Z. officinale*–mediated CuO NPs as promising candidates for therapeutic and biomedical applications.

## Clinical trial number

Not applicable.

## Introduction

 The field of nanotechnology is establishing itself as a transformative tool utilized across several domains, encompassing drug delivery systems for antibacterial and anticancer treatments, diagnostic tools for tumor identification in healthcare, and agricultural methods for plant protection and nutrition enhancement [1–6]. Nanoparticles, particularly metals and metal oxide nanoparticles with diameters under 100 nm, have proven useful in treating infectious disorders due to antibiotic resistance caused by resistant microbes [7–11]. Scientists have demonstrated that several metal-based nanomaterials, including copper, silver, zinc, gold, and titanium, display a wide range of antibacterial efficacy against diverse types of bacteria [12–17]. Multiple methods exist for nanoparticle synthesis, particularly the method known as green synthesis, offering numerous benefits such as lower temperature and pressure requirements, along with the utilization of less hazardous chemicals, rendering it both cost-effective and environmentally sustainable [18–20]. Moreover, it entails the use of biological systems and their by-products, which collectively facilitate the efficient large-scale production of nanoparticles while minimizing toxicity to both humans and the environment [21–25].

Among the various resources utilized in nanoparticle manufacturing, plants exhibit significant potential [26, 27]. Copper is a metal that is more readily available and less expensive than other metals like silver and gold [28, 29]. Nanoparticles synthesized using the green method include CuO NPs, which are characterized by a substantial surface-to-volume ratio, endowing them with optical, catalytic, and magnetic properties superior to those of bulk materials [30, 31]. They are utilized in superconducting materials, batteries, gas sensors, pharmaceuticals, cosmetics, and as catalysts for the hydrogenation of organic dyes [32]. Various natural resources, including plants, microorganisms, and fungi, are employed for the synthesis of CuO NPs [33, 34]. Plant extracts contain an assortment of phytochemicals and metabolites, including vitamins, carbohydrates, phenolics, and flavonoids, that can function as reducing and stabilizing-agents, converting Cu²⁺ to CuO NPs [35, 36].

Ginger (*Zingiber officinale*), a prominent medicinal plant with a diverse phytochemical profile, has garnered significant attention as both a spice and an herbal remedy. Flavonoids, phenolic compounds, and terpenoids constitute only a fraction of its bioactive constituents [37]. Ginger has long been extensively utilized in ethnomedicine to feed the treatment of various diseases. Ginger formulations are predominantly utilized in modern phytotherapy to relieve nausea. Additionally, they demonstrate anti-inflammatory, analgesic, and metabolic effects in clinical scenarios, including dysmenorrhea, type-2 diabetes, high cholesterol levels, and obesity [38]. Ginger extract serves as a promising agent for nanoparticle synthesis due to its constituents, including gingerol and zingerone, which possess potent reducing activities [39]. Consequently, ginger is rich in antioxidants; therefore, the biological molecules present in the extract play an important role in reducing metal ions and stabilizing metallic nanoparticles [40].

CuO NPs are especially valued for their efficacy in scavenging oxygen-containing free radicals [41]. CuO NPs, along with other highly ionic metal oxide nanoparticles, is considered important due to their distinctive crystal structures and elevated surface area [42]. Recent research has highlighted the potential uses of CuO NPs produced via green-methods, including drug delivery [43], antibacterial [44], anticancer [45], anti-inflammatory, antidiabetic [46, 47] and antioxidant [48] activities. These nanoparticles exhibit exceptional antibacterial efficacy, rendering them advantageous for wound dressings and antiseptics [49].

This research aims to examine the biological applications of CuONPs derived from *Z. officinale* extract, focusing on their antibacterial, antioxidant, antibiofilm, antiviral, anticancer, anti-inflammatory, and antidiabetic properties. A phytochemical investigation and GC-MS analysis were conducted on the *Z. officinale* extract. The resulting CuO NPs characterized using FT-IR, TEM, XRD, EDX, and SEM techniques.

## Materials and methods

### Plant material collection and authentication

Fresh rhizomes of *Zingiber officinale* (ginger) were obtained from the Egyptian Ministry of Agriculture in Cairo, Egypt, in October 2024. The plant material was identified and authenticated by a senior botanist at the Department of Botany and Microbiology, Faculty of Science, Al-Azhar University. The experimental work, including extraction, synthesis, and all bioactivity assays, was conducted between November 2024 and May 2025.

### GC-MS analysis

The GC-MS study was performed in accordance with the published methods [50]. With helium gas as a barrier and a steady flow of 1 mL/min., GC/MS detected using an ionization-energy of 70 eV. 280 °C was the temperature selected for the injector and MS-transfer line. For two minutes, the oven was set to achieve and maintain 50 °C. Following that, it was programmed to increase by 7° to 150 °C /min., then by 5 °C /min. to 270 °C, and lastly to hold it for an additional two minutes. Ultimately, it was set to increase at 3.5 °C per min. to reach the target Temp. of 310 °C (hold for 10 min.). Every component that was discovered was measured using a relative peak area percentage. Comparing the mass-spectra and irrelative retention time with the GC/MS apparatus’s NIST, WILLY library data allowed for a tentative identification of the chemicals.

### Phytochemical assay

*Z. officinale* underwent phytochemical examination to qualitatively and quantitatively assess the existence of various chemical components, as outlined in Harborne’s studies [51], and Sofowara [52]. Total phenolic acids, total flavonoid, total tannins, total flavonol, total steroid, total alkaloid and total saponin was performed as described in previous study [53].

### Biosynthesis of CuONPs using *Zingiber officinale*

The *Z. officinale* was purchased from the nearest market inside Egypt. Some distinct cleans in flowing water were employed to eliminate any lingering depicts of dust or dirt coming from the outermost layer. Subsequently, we incorporated 100 mL of dH_2_O towards 20 g of rhizome that had been segmented into smaller segments. The mixture was maintained at 100 rpm and 60 ◦ C for a duration of 60 min. After a 10-minute centrifugation at 1,000 rpm, the resulting supernatant was gathered to conduct the environmentally friendly production of CuO NPs.

The process of synthesizing CuO NPs involved the incorporation of copper acetate monohydrate into a water-based extract derived from *Z. officinale*. In summary, ten g of salt was mixed in 100 mL of the prepared extract as well as stirred for 2 h at 50 °C. Following the reaction process completed, the solution underwent centrifugation for ten minutes at 10,000 rpm to allow the disintegrated form precipitates to settle down, and the CuO NPs were taken out as a pellet. The resulting pellet underwent three washes with water that had been distilled and two washes with ethanol through centrifugation to enhance purification, followed by a drying process at 40 °C for 24 h. Then the CuO NPs underwent calcination at 200 °C for 3 h. to eliminate additional impurities and achieve a crystal-like appearance.

### Characterization

The objective of this investigation was to identify the different functional groups present on the nanoparticles. Fourier-transform infrared spectroscopy (FTIR) was used to obtain the absorption spectra of the CuO NPs. This technique identifies functional groups by measuring their interaction with infrared radiation. Pellets containing the synthesized CuO NPs were prepared and analyzed using an Agilent FTIR spectrometer (Cary 630), and the spectra were recorded. XRD analysis was performed to determine the crystalline structure of the biologically synthesized CuO NPs. This technique is useful for estimating the crystallinity and phase of NPs and provides an estimate of the particle size using the Scherrer equation. The X-ray diffraction data were obtained *using a* Philips PANalytical X’pert PRO diffractometer with a step size of 0.02° and a scanning range from 10° to 110°. A glass slide was coated with a thin layer of the synthesized CuO NPs powder and placed inside the XRD chamber. The size and morphology of the NPs were examined using a JEOL 1010 TEM. A drop of the sample was applied to a carbon-coated copper TEM grid and allowed to adsorb completely. Any excess liquid was carefully removed by blotting with filter paper prior to examination. The surface morphology and elemental composition of the CuO NPs were examined using SEM coupled with a JEOL JSM-6510 LV energy dispersive X-ray spectroscopy (EDS) system.

### Antimicrobial activity

The antibacterial efficacy of the phytosynthesized CuO NPs was investigated against several pathogenic bacterial strains, including *E. coli*,* K. pneumoniae*,* P. aeruginosa*,* E. faecalis*,* S. aureus*, and *B. subtilis*. The antibacterial activity was first assessed using the agar well diffusion method [54]. Briefly, the microorganisms were grown on nutrient agar for 24 h at 35 ± 2 °C. Then, bacterial suspensions were swabbed evenly onto the surface of Mueller-Hinton agar plates, and wells measuring 6 mm in diameter were created in each plate. Subsequently, 100 µL of the CuO NPs suspension (at a concentration of 1 mg/mL) was introduced into each well. The plates were then refrigerated for approximately one hour to allow for diffusion and subsequently incubated at 37 °C under optimal conditions. Following incubation, the resulting inhibition zones around the wells were measured [55].

The MIC was defined as the lowest concentration of an antimicrobial agent that inhibits visible bacterial growth. The MIC of the NPs was determined using a broth microdilution assay. Two-fold serial dilutions of CuO NPs (0–1000 µg/mL) were prepared in Mueller-Hinton (MH) broth in a microtiter plate. Then, 100 µL of a standardized bacterial inoculum (a 24-hour culture adjusted to 0.5 McFarland standard turbidity) was added to each well containing 100 µL of the NP-supplemented broth. The plate was incubated at 37 °C for 24 h. After incubation, 20 µL of resazurin dye was added to each well, and the plate was kept in the dark. A change in the dye color to pink indicated bacterial growth and, consequently, the absence of antibacterial activity at that concentration. To determine the minimum bactericidal concentration (MBC), samples from wells with nanoparticle concentrations equal to or greater than the MIC were subculture onto MH agar plates and incubated for 24 h. The MBC was defined as the lowest concentration of CuO NPs that completely prevented bacterial growth on the agar subculture [56].

### Biofilm inhibition assay

To determine if CuO NPs could prevent or reduce biofilm formation by *S. aureus* and *E. coli*, which are major biofilm-forming strains, the MTP method was used with specific modifications [8]. A flat-bottomed microtiter plate with tryptic soy broth medium and 1% glucose was prepared with different concentrations of CuO NPs (ranging from 6.25 µg/mL to 100 µg/mL). An inoculum with a density of 1.5 × 10^8^ CFU/mL was obtained from overnight cultures of test organisms, diluted to 1:100, and incubated at 37 °C for 48 h in the microtiter plate. After incubation, planktonic cells were removed from all microtiter plate wells without disturbing the biofilm. The wells were washed at least three times with PBS to remove unattached cells. Equal volumes of 200 µL of 95% methanol were added to each well to fix the biofilm. Next, crystal violet (CV) dye was added to the wells and kept at room temperature for approximately 20 min, followed by rinsing with d H₂O. Finally, an Olympus CK40 inverted microscope at 150x was used to analyze and photograph the CV-stained biofilm. To quantify biofilm growth, glacial acetic acid was added to each well and the absorbance was measured at 540 nm using a microplate reader (Tecan Elx800). The results from treated and untreated wells were compared.

### Antioxidant assay

The antioxidant capability of generated CuO NPs was measured by employing DPPH (2,2-diphenyl-1-picrylhydrazyl, Sigma-Aldrich, USA). At varying quantities 3.9 to 1000 µg/mL that were dispersed in Milli-Q H_2_O to produce different doses. Next, add 1 mL of the resulting solution into a test tube with 1 mL of DPPH within methanol and 450 µL of Tris-HCl buffer (pH 7.4, 50 mM), mix thoroughly, and remain in dark conditions at 37 °C for 0.5 h pursuant to using 100 rpm agitation. Ascorbic acid (positive control) was used in a different set of trials under identical settings and dosages. The negative control—DPPH and Tris-HCl buffer without CuO NPs or ascorbic acid—was also incubated with the identical circumstances. The color’s absorbance was 517 nm after incubation. Calculating free radical scavenging via these equation [57]:

$$\rm RSA\:(\%) = [ (A{\_}control - A{\_}sample) / A{\_}control ] \times 100$$

### Cytotoxic and anticancer assay

MTT assays investigated CuO NPs cytotoxicity against normal Vero (ATCC CCL-81) cell lines as well as anti-cancer activity against Mcf7 (ATCC HTB-22) and Caco2 (ATCC ATB-37), Science way Co., Egypt [58]. For a complete monolayer sheet, put 100 µL/well of 10^5^ cells/mL to each well of cell culture plate followed by incubation over 24 h at 35˚ C. Each individual cell monolayer was washed twice using cleaning fluid after a convergent sheet of developed cells in MTP. The sample examined was diluted twice in RPMI with 2% serum. Each solution was tested for 0.1 mL in a separate effectively, keeping three wells as upkeep medium standards. Following that, incubation and tested. Cell loss of volume, rounding, granulation, or monolayer disintegration were used to assess cytotoxicity. A 5 mg/mL MTT solution (Bio Basic, Canada) and 20 µL of sample was added to each well. After agitating for 5 minutes at 150 rpm, the samples were incubated at 37˚C with 5% CO_2_ about 4 h to initiate MTT metabolism [59].

The optical density was measured at 560 nm. Cell viability was calculated using the following equation:

$${\rm{Cell}}\,{\rm{Viability }}\left( {\rm{\% }} \right){\rm{ = }}\left( {{\rm{OD\_sample / OD\_control}}} \right){\rm{ \times 100}}$$

### Assessment of CuO NPs’ antiviral efficacy

CuO NPs were dissolved in serum-free DMEM medium before being used at concentrations of 125, 62.5, 31.25, and 15.62 µg/ml. Vero cells were seeded in MTP; upon achieving 80% confluency, the medium was removed, and the wells were washed using PBS. Subsequently, 50 µl of the tested virus and 100 µl of different concentrations of CuO NPs were added to 50 µl of DMEM. This resulted in a final volume of 200 µl per well, with the CuO NPs at the intended final concentrations of 125, 62.5, 31.25, and 15.62 µg/ml for the dose-response assessment. Cultures with the virus served as the viral control (virus + DMEM), whereas the Vero cells alone acted as the cell control. The plate was then incubated for about 24 h at 37 °C in 5% CO₂.

In a separate set of experiments, monolayers were infected with 50 µl of virus for 2, 6, and 8 h at 35 °C. Following the incubation periods of 2, 6, and 8 h, 100 µl of CuO NPs were added to the culture, which was then incubated under the same conditions with 5% CO₂. In another approach, the virus was pre-incubated with varying concentrations of CuO NPs for about two hours, after which 0.2 ml of the virus-nanoparticle mixture was added to the monolayers. Finally, the plates were incubated under the same conditions as described above [60].

### Assessment of CuO NPs’ anti-diabetic efficacy

#### α-amylase assay

The study was conducted employing the 3,5 dinitrosalicylic acid (DNSA) technique. The CuO NPs were introduced to the buffer composed of 0.03 M NaH_2_PO_4_/Na_2_HPO_4_ and 0.007 M NaCl at pH 7.0 to facilitate additional dissolution, shortly after initial dissolution in 10% dimethyl sulfoxide (DMSO). Approximately 200 µL of the CuO NPs were mixed with a concentration of 2 units/mL of α-amylase and thereafter incubated at 32 °C for 9.0 min. Subsequently, the mixture was treated with 200 µL of a 1% (w/v) aqueous solution of starch and allowed to stand for 3 min. Upon completion of this reaction, 0.2 mL of DNSA (comprising 24.0 g of sodium potassium tartrate tetrahydrate dissolved in 16 mL of 2 M NaOH and 40 mL of 96 mM 3,5-dinitrosalicylic acid) was heated for about 10 min at 95 °C. The resulting solution was allowed to cool to 20 °C prior to dilution with 5.0 mL of d H₂O. The absorbance was then measured at a wavelength of 540 nm using a MiltonRoy310 spectrophotometer [61].

#### α-glucosidase investigation

A glucosidase suppression experiment was performed to evaluate the α-glucosidase inhibitory capacity of CuO NPs by applying a modified method from Zafar et al. [62]. To solubilize α-glucosidase, 100 mL of phosphate buffer (pH 7) was combined with 200 mg of bovine serum albumin. Following a 5-minute incubation, 490 µL of phosphate buffer (pH 7) and 250 µL of **(**p-nitrophenyl-β-d-glucopyranoside) (5 mM) were combined and incubated at about 37 °C for 15 min. Thereafter, CuO NPs were added to 0.25 mL of α-glucosidase and incubated under the same conditions. The process was halted with a 2 mL solution of Na_2_CO_3_ (200 mM), and the absorbance was measured at 400 nm. The investigation was conducted three times to reduce error, using acarbose as a positive control [63].

### Statical analysis

Experiments were performed in triplicate or more (*n* ≥ 3), and data are reported as mean ± SD. Statistical analysis used one-way ANOVA with Tukey’s post-hoc test or two-way ANOVA when appropriate, conducted in GraphPad Prism 8. Significance was set at *p* < 0.05.

## Results and discussion

### GC-MS analysis

Mass spectra were included in computer search user-generated reference libraries to obtain the identification [50, 64, 65]. Various compounds were identified in *Z. officinale* rhizome chloroform extract by GC-MS (Table 1). Table 3 shows the major components present in GC-MS examination of *Z. officinale* rhizome chloroform extract were Isoauraptene (13.31%), 8-(2,3-Dihydroxy-3-methylbutyl)-7-m ethoxy-2 H-chromen-2-one (10.66%), dl-à-Tocopherol (7.36%), n-Hexadecanoic acid (5.96%), Osthole (5.57%), Linoleic acid ethyl ester (5.03%), and stigmast-5-en-3-ol, (3á,24s)- (4.49%). These compounds are responsible for various pharmacological actions like antibacterial [66], antioxidants, antibiofilm, and cytotoxicity were attributed to these chemical ingredients [67].

**Table 1:** GC-MS chromatogram of *Z. officinale* chloroform extract

No.	*R*_t_	Area %	M.W.	M.F.	Identified compounds
1	6.49	0.19	170	C_10_H_18_O_2_	2-Furan-methanol,5-Ethenyltetrahydro-à,à,5-tri-methyl-,cis
2	8.63	0.18	154	C_10_H_18_O	L-à-Terpineol
3	10.39	0.17	196	C_12_H_20_O_2_	1,6-octa-dien-3-ol, 3,7-di-methyl-, acetate
4	14.54	0.31	204	C_15_H_24_	Caryophyllene
5	17.82	0.65	222	C_15_H_26_O	1,6,10-Dodecatrien-3-ol, 3,7,11-tri-methyl-, (E)-
6	19.09	0.11	242	C_15_H_30_O_2_	Pentadecanoic acid
7	21.21	0.29	390	C_24_H_38_O_4_	Phthalic acid, butyl dodecyl ester
8	22.24	0.67	228	C_14_H_28_O_2_	Tetradecanoic acid
9	26.33	5.96	256	C_16_H_32_O_2_	n-Hexadecanoic acid
10	26.90	2.66	284	C_18_H_36_O_2_	Hexa-decanoic acid, ethyl-ester
11	28.44	5.57	244	C_15_H_16_O_3_	Osthole
12	28.74	0.14	282	C_18_H_34_O_2_	Oleic Acid
13	29.82	5.03	308	C_20_H_36_O_2_	Linoleic acid ethyl -ester
14	29.88	3.41	306	C_20_H_34_O_2_	9,12,15-Octa-decatrienoic acid, ethyl-ester, (Z, Z,Z)-
15	29.99	13.31	260	C_15_H_16_O_4_	Isoauraptene
16	31.33	0.16	237	C_13_H_19_NO_3_	5-Iso-quinolinol, 1,2,3,4-tetra-hydro-6,7-dimet hoxy-1,2-di-methyl-, (S)-
17	33.07	10.66	278	C_15_H_18_O_5_	8-(2,3-Di-hydroxy-3-methylbutyl)-7-m ethoxy-2 H-chromen-2-one
18	35.31	1.42	390	C_24_H_38_O_4_	Diisooctyl phthalate
19	37.73	0.50	338	C_21_H_38_O_3_	Glycidyl oleate
20	40.09	2.24	410	C_30_H_50_	Squalene
21	41.66	0.10	444	C_30_H_52_O_2_	Tri-cyclo[20.8.0.0(7,16)]tri-acontane,1(22),7(16)-diepoxy-
22	42.82	7.36	430	C_29_H_50_O_2_	dl-à-Tocopherol
23	44.23	2.36	412	C_29_H_48_O	Stigmasterol
24	44.98	2.78	388	C_28_H_24_N_2_	2,9-bis(2’,6-di-methylphenyl)- 1,10-phenanthroline
25	45.07	4.49	414	C_29_H_50_O	stigmast-5-en-3-ol, (3á,24s-

### Phytochemical study

Qualitative investigation of the phytochemical composition of dried rhizome of ginger reveals that there are various phytochemical substances as carbohydrates, glycosides, tannin, alkaloids, saponins, steroids, flavonoids, phenols, terpenoids, amino acids, and anthraquinones, had been identified by making chemical analysis as preliminary phytochemical screening of *Z. Officinale* rhizome. Phlobatannins were not detected. The result is shown in Table 2.

The findings from the phytochemical analysis of ginger indicated the presence of flavonoids. Flavonoids exhibit antioxidant properties and promote healthy blood circulation. They also contribute to the fortification of the capillary walls. These compounds are occasionally identified as phytoestrogens. Phytoestrogens are linked to alleviating menopausal symptoms, decreasing osteoporosis risk, enhancing blood cholesterol profiles, and reducing the likelihood of specific hormone-related cancers [68]. Additionally, polyphenols and other reducing sugars are also phytochemical components of *ginger rhizome* [69].In the field of medicine, polyphenols are believed to be linked to preventing aging and preventing the spread of cancer [68].Other researchers’ reports indicate that phytochemical screening of certain medicinal plants has identified the presence of alkaloids, carbohydrates, saponins, flavonoids, and phenolic compounds that are linked to antimicrobial activities and therapeutic properties against pathogens, aligning with the findings of this study.

**Table 2:** Preliminary Phyto-chemical Screening of *Z. Officinale*

Active compounds	Inference
Carbohydrates	+
Amino acids	+
Phenols	+
Tannins	+
Phlobatannins	-
Flavonoids	+
Saponins	+
Glycosides	+
Alkaloids	+
Terpenoids	+
Steroids	+
Anthraquinones	+

The phytochemical compositions of *Z. officinale* rhizome are shown in Table 3. In *Z. officinale*, the total flavonoid content was 391.75 ± 1.61 mg QE/g dry weight, while flavonols reached 124.39 ± 0.9 mg RTE/g, phenolic acids 136.65 ± 0.7 mg GAE/g, and tannins 88.08 ± 0.63 mg TAE/g. The extract also contained 3.67 ± 0.22 mg/100 g of alkaloids, 2.75 ± 0.24 mg/100 g of saponins, and 1.05 ± 0.09 mg/100 g of steroids. Our findings have similarities to those of [70].

**Table 3:** Quantitative phytochemical-analysis of *Z. Officinale* rhizome

Parameters	Z. Officinale rhizome
Total-flavonoids	391.75 ± 1.61 mg QE /g
Total-flavonols	124.39 ± 0.9 mg RTE /g
Total-phenolic acids	136.65 ± 0.7 mg GAE /g
Total-tannins	88.08 ± 0.63 mg TAE /g
Total-alkaloids	3.67 ± 0.22 mg/100 g
Total- saponins	2.75 ± 0.24 mg/100 g
Total -steroids	1.05 ± 0.09 mg/100 g

### Characterization of CuO NPs

**Fig. 1:**
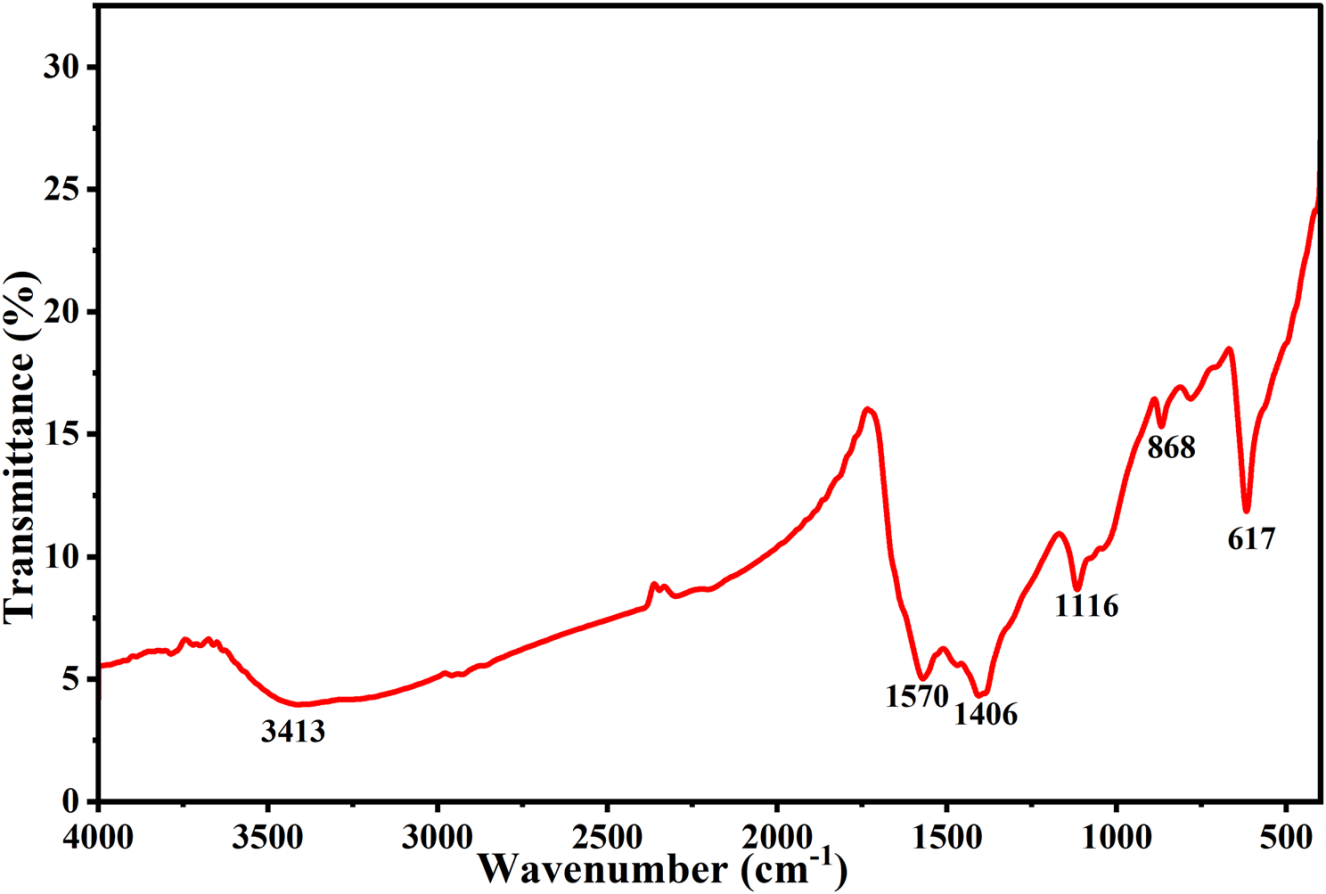
FTIR spectra of Biosynthesized CuO NPs

The functional groups directing the synthesis and stabilization of CuO NPs were identified using FTIR analysis. FTIR analysis confirmed that the material did indeed include several functional groups. To identify possible biomolecules with bio-reducing capabilities in the plant extract, FTIR analysis was used. Prominent bands were seen in the spectra of CuO NPs at 3413, 1570, 1406, 1116, 868 and 617 cm − 1, respectively (Fig. 1). The FTIR analysis of CuO NPs synthesized using a plant extract reveals a broad absorption band near 3413 cm⁻¹, attributed to hydroxyl (O–H) stretching vibrations, indicating the presence of alcohols or phenolic compounds from the extract [71]. Additionally, a distinct peak at 1570 cm⁻¹ is associated with the C = O stretching vibrations of carboxylic groups, suggesting that bioactive molecules in the extract were involved in both the reduction and stabilization of the nanoparticles [72]. The aromatic amines’ C-N stretching vibrations were represented by the peaks at 1406 cm − 1 [73]. The C-O stretching vibrations were represented by the peaks at 1116 cm − 1 [74]. The absorption bands at 868 and 617 cm–1 is detected in biosynthesized CuO NPs and are assigned to the metal oxide [75, 76]. Therefore, the FTIR data showed that the extract’s biomolecules, such as phenolic chemicals, flavonoids, and terpenoids, may be responsible for the extended stability of CuO NPs by capping and reducing them. Furthermore, the drop in the intensity of these functional group bands following a decrease of Cu2 + ions indicates the participation of phenol and flavonoid sites in the binding process of CuO NPs [77]. The outcomes jointly demonstrate the effective reduction of CuO to CuO NPs. The results we report align alongside earlier research published regarding the properties of green-synthesized CuO NPs [78].

**Fig. 2:**
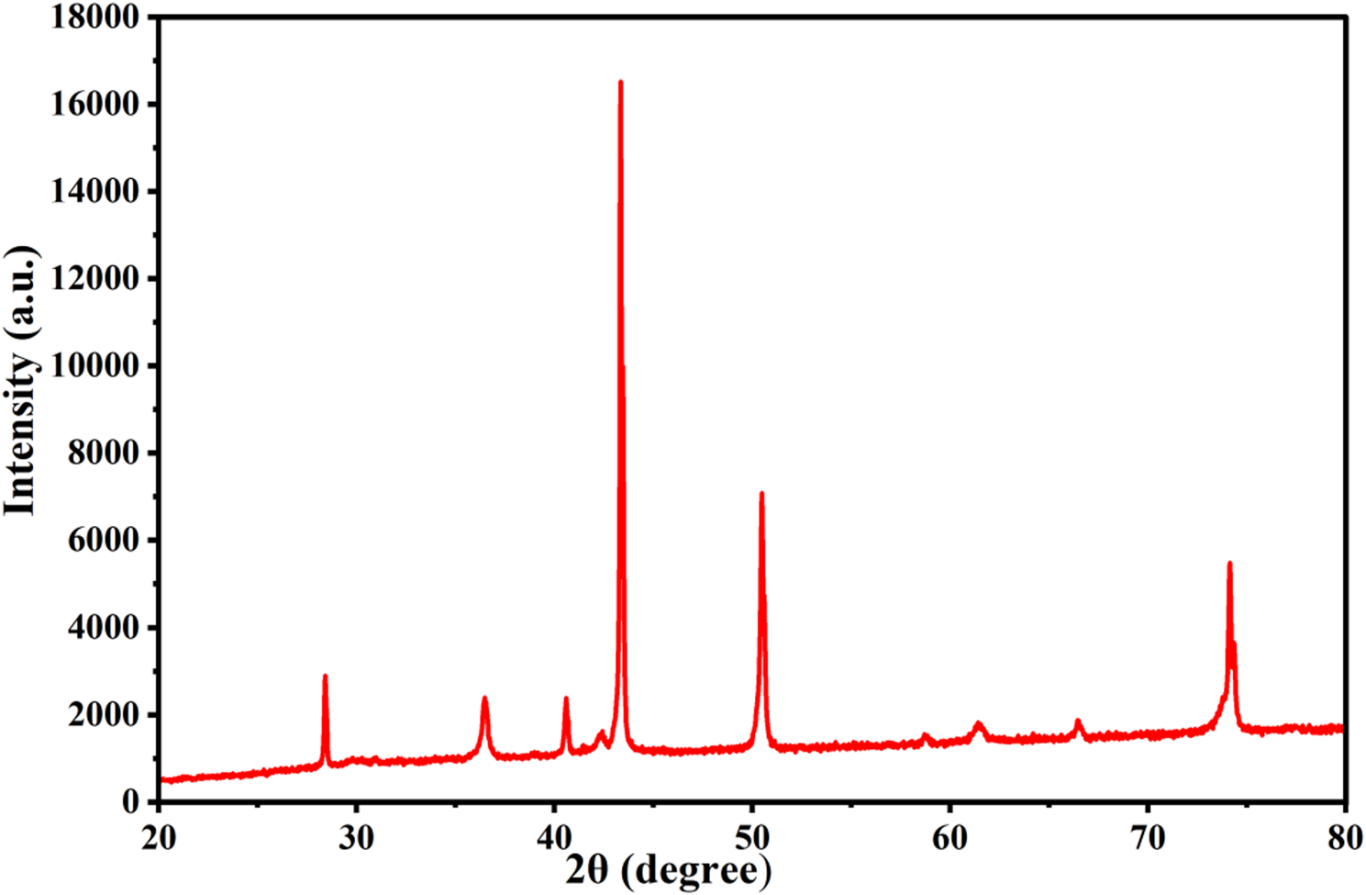
XRD spectra of Biosynthesized CuO NPs

The results presented in Fig. 2, for XRD-pattern revealed distinct, intense-peaks at the following 2θ-angles: 28.3°, 36.4 °, 40.5°, 43.3°, 50.4°, 58.6°, 61.3°, 66.4°, and 74.1°, which correspond to the crystallographic planes 110, 002, 111, 202, 020, 202, 113, 311, and 222, respectively. The observed diffraction peaks closely correspond to those reported in JCPDS-Card No. 45-0937, confirming the formation of crystalline CuO, consistent with the standard pattern for bulk copper(II) oxide. The exceptional purity of the produced CuO NPs is confirmed by the lack of extra peaks from other phases and the consistency of all detected diffraction peaks with a monoclinic crystal-structure. Furthermore, the well-crystalline structure of the nanoparticles is indicated by the distinct and crisp CuO reflections in the XRD-patterns [79]. The detected peak within the 2θ spectrum from 35–39° suggests the synthesis of CuO NPs, consistent with earlier findings [34]. The lack of extra peaks in the XRD image validated the outstanding purity of the generated CuO NPs, consistent from the EDX examination. The data gathered aligned well with multiple studies on green-synthesized CuO NPs [76, 80]. Our findings disclose revealed the crystalline state of CuO NPs exhibited similar maximum indices that described in the study done by Taghavi et al. [81].

**Fig. 3:**
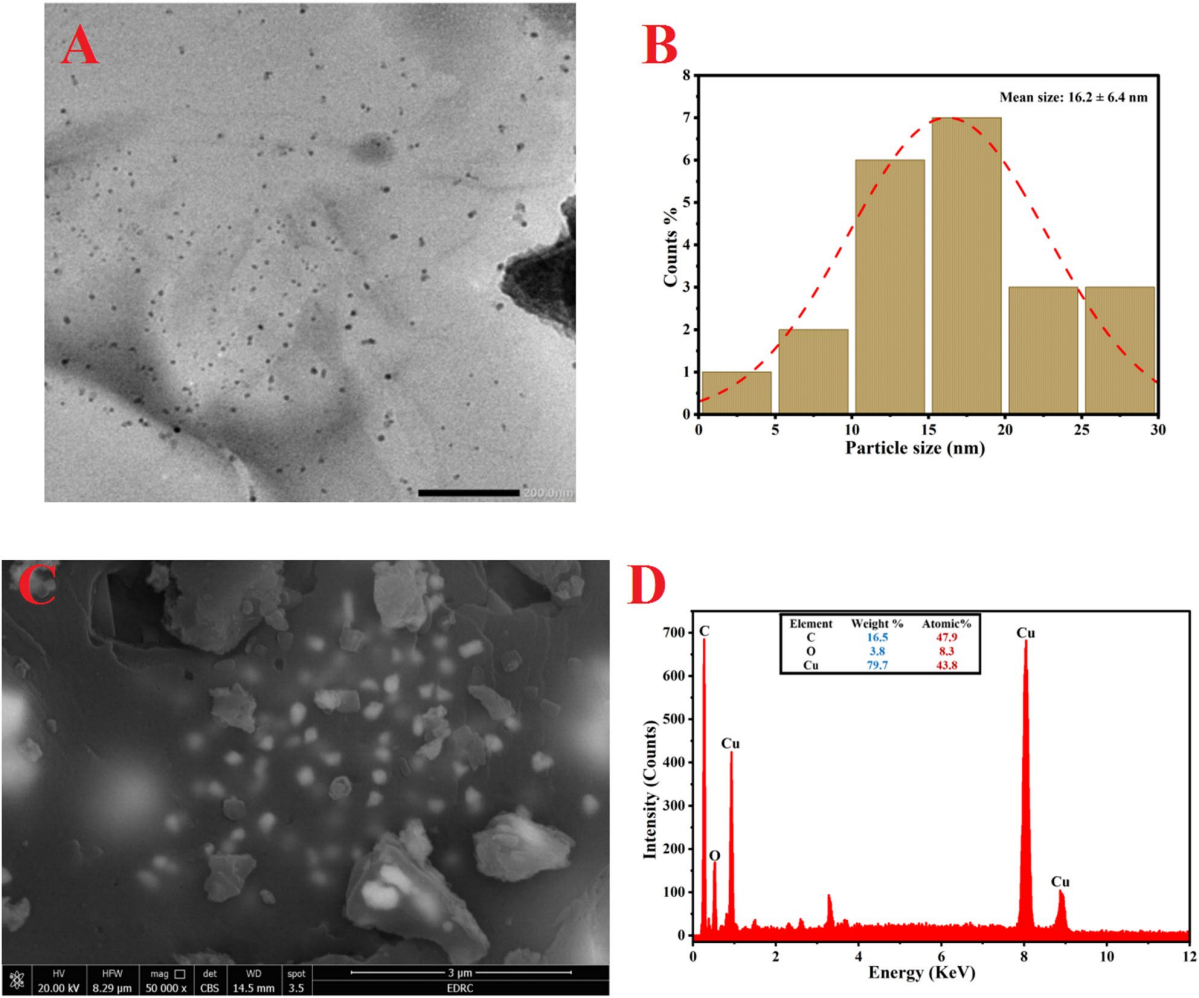
TEM(**A**), PSA(**B**), SEM(**C**) and EDX(**D**) analysis of Biosynthesized CuO NPs

The topological properties, such as dimensions, forms, accumulations, and fundamental patterning of green-synthesized CuO NPs, are essential factors influencing their biological activity. TEM, SEM, and EDX investigations are valuable methodologies for examining these properties. TEM analysis confirmed the spherical morphology of the biosynthesized CuO NPs (Fig. 3A). Particle size distribution analysis revealed a size range of 4 to 28 nm, with an average diameter of 16.2 nm (Fig. 3B). The histogram indicates a relatively narrow size distribution, which is characteristic of a homogeneous synthesis process. In a comparable work, the mean particle size for the sphere CuO NPs synthesized employing the water-soluble extract of *Annona muricata* L was between 16 and 31 nm [82]. The biological properties of nanostructures have a direct relationship to their sizes and shapes. The cytotoxic capacity of CuO NPs varies according to diameters ranging from 4 nm to 24 nm. Reports indicate that CuO NPs measuring 24 nm exhibit much greater toxicity to A549 adenocarcinoma cells than those measuring 4 nm, despite the latter’s superior ability to dissolve poisonous ions (Cu2+) more rapidly than their larger counterparts [83]. The antibacterial efficacy of rod and platelet-shaped CuO NPs synthesised from Aloe vera water extract against *E. coli* as well as Staphylococcus aureus was superior to that of spherical forms [84]. The variance in engagement may be attributed to the elevated surface energy associated surface-active regions of various fundamental morphologies [85]. Additionally, the morphological characteristics of plant-derived CuO NPs were examined by SEM analysis (Fig. 3C). The CuO NPs exhibited a smooth surface and spherical morphology, organized lacking agglomeration. The spherical morphology of synthesized CuO NPs, utilizing extracts from mint buds or orange peel, was observed through SEM investigation [86]. Certain aggregations in SEM images result from coating agents derived from plant material, potentially enlarging their size as observed in SEM in contrast to TEM examination. The chemical components of green synthesized CuO NPs were analyzed by EDX, as illustrated in (Fig. 3D). EDX analysis showed that the biosynthesized product mainly consisted of copper and oxygen. The 0.5 KeV signal represented oxygen, while the peaks at 1.0, 8.0, and 9.0 KeV were due to copper. Quantitative results revealed weight percentages of 16.5% for oxygen and 79.7% for copper, with atomic percentages of 47.9% and 43.8%, respectively (Fig. 3c). The dispersion of phytochemicals from the plant’s water-based extract that covered the CuO NPs surface is what caused this signal [86]. As mentioned in previous study found that the EDX spectrum of plant based CuO NPs showed weight fractions of 20.1% as well as 79.9%, correspondingly [75]. The EDX analysis of CuO NPs synthesized from the *Azadirachta indica* leaves aqueous-extract indicated the existence of Cu (65.33%) and O atoms(34.67%) weight percentages [87].

### Antibacterial sensitivities assessment of bio fabricated CuO NPs

**Fig. 4:**
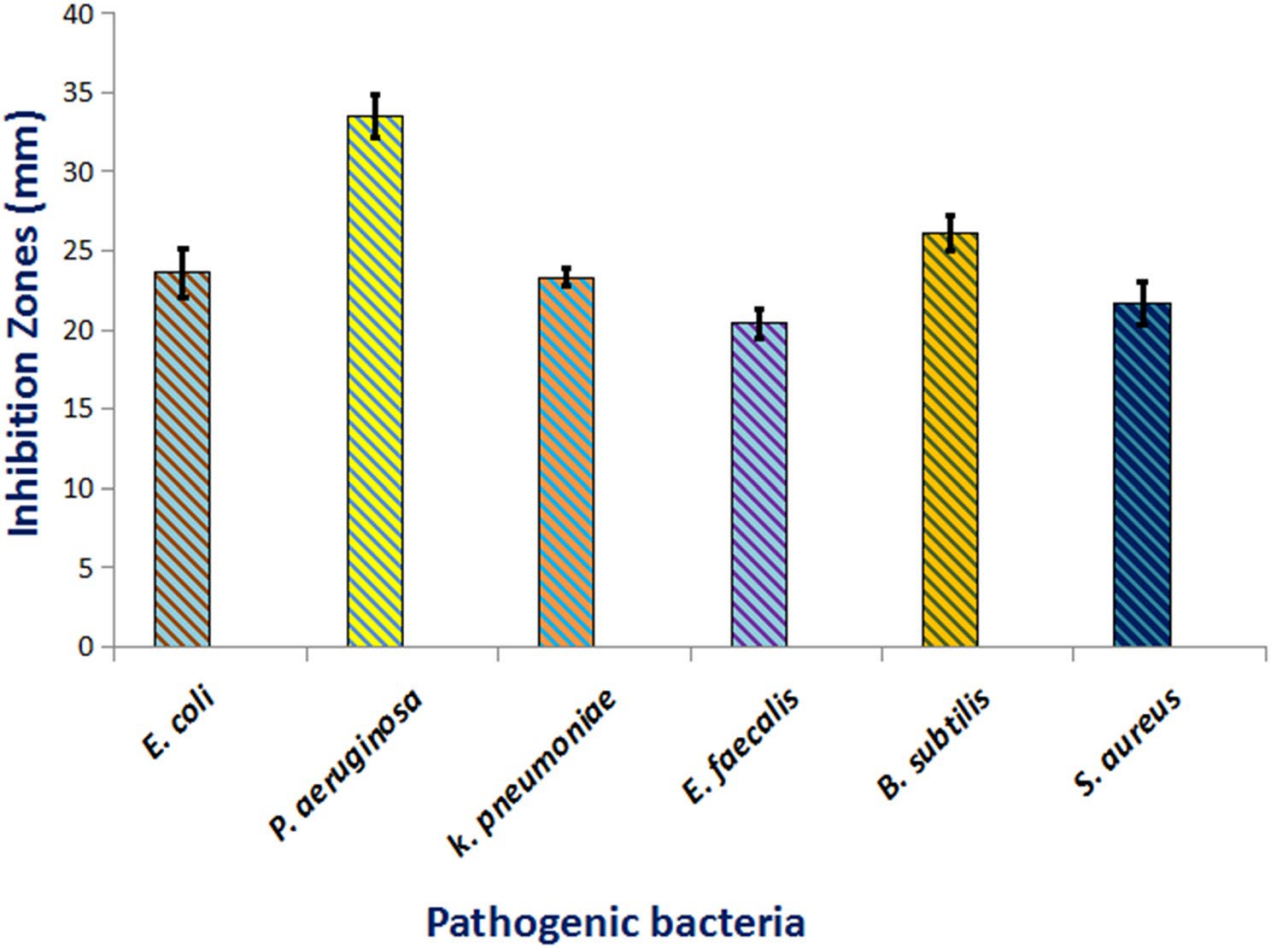
Antibacterial efficacy of CuO NPs versus various pathogenic bacteria

The efficacy of CuO NPs as antibacterial was investigated via the agar well diffusion technique, determining the diameter of restrictive zones versus bacterial strains. The findings in Fig. 4 demonstrated a dose-dependent inhibitory effect of CuO NPs, with the width of the suppressive zones rising as the quantity of the nanoparticles rose. The dimension of the suppression zone ranged from 20 to 33.5 mm. *P. aeruginosa* was recorded with the highest antibacterial effect (33.5 mm) at a dose of 1000 µg/ml, after *B. subtilis* (26.1 mm), whereas the smallest inhibitory zone was reported against *E faecalis* (21.4 mm). The effectiveness of the antibacterial of CuO NPs was assessed by determining the MIC and MBC through the broth--based microdilution technique. The result indicates that *B. subtilis* had the lowest MIC-value of 50 µg/ml, meanwhile the MIC values for the other strains ranged from 600 to 100 µg/ml. Correspondingly, the MBC values were 300, 400, 200, 600, 100, and 800 µg/ml for *P- aeruginosa*,* E-coli*,* K- pneumoniae*,* E-faecalis*,* B -subtilis*, and *S- aureus*, respectively. Bio fabricated CuO NPs may exhibit varying antibacterial activity towards certain bacteria, contingent upon the synergy of biologically active metabolites that contribute to the production process. Padil and Cernik investigated the antibacterial effectiveness of CuO NPs, reporting MIC values of 120 µg/ml for *Staphylococcus aureus* and 10 µg/ml for *Escherichia coli* [88]. Conversely, other studies reported MIC values of 62.5, 31.25, and 250 µg/ml for *S. aureus*,* E coli*,* and K pneumoniae*, accordingly [89]. Naturally occurring CuO NPs derived from the extract of *Pterocarpus marsupium* exhibited suppressive zones of 25 mm versus *K pneumoniae*, 24 mm against *E coli*, and 20 mm versus *S. aureus* during a 24-hour incubation period [90]. A comparable study demonstrated the notable antimicrobial properties of naturally synthesized CuO NPs against *B subtilis*,* S aureus*,* P aeruginosa*,* E coli*,* Acinetobacter sp*. at higher levels (170 ppm) in contrast to their activity at diminished amounts (100 and 50 ppm) [91]. The disparity in MIC values against harmful bacteria may be ascribed to factors such as synthesis technique, dimensions, morphology, agglomeration percentages, as well as charge on the surface [92]. The generation of harmful ions, namely Cu^2+^, after the degradation of nanoparticles inside bacterial cells, is regarded as the primary mechanism behind the inhibitory effect of CuO NPs. Toxic ions released interact via the thiol group of proteins, hence inhibiting their activity [93].

In addition to the plant-mediated CuO NPs is well-documented, demonstrating efficacy against pathogens such as *Pseudomonas aeruginosa* and *Proteus vulgaris* [94]. This enhanced antibacterial activity, as seen in particles derived from *Allahabad Safeda*, is largely due to their unique surface properties and high chemical reactivity, which promote effective microbial contact [95]. The diminutive size of these NPs further potentiates their action by facilitating greater interaction with bacterial cells. The primary mechanism involves an assault on the cell envelope, where CuO NPs induce structural alterations that compromise membrane integrity, ultimately leading to cell disintegration and lysis [96]. An additional strategic action involves the disruption of bacterial quorum-sensing by potent phytometabolites associated with the NPs, which can suppress the expression of virulence factors and prevent outbreaks. The release of copper ions (Cu²⁺) also plays a critical role; these cations electrostatically adhere to and permeate the bacterial membrane, significantly enhancing bactericidal efficacy [97].In summary, Phyto fabricated CuO NPs deploy a multi-mechanistic antimicrobial strategy encompassing direct membrane damage, ROS-induced oxidative stress, interference with DNA processes, and quorum-sensing inhibition, making them potent agents against resistant pathogens [98].

### Antibiofilm efficacy of CuO NPs

**Fig. 5:**
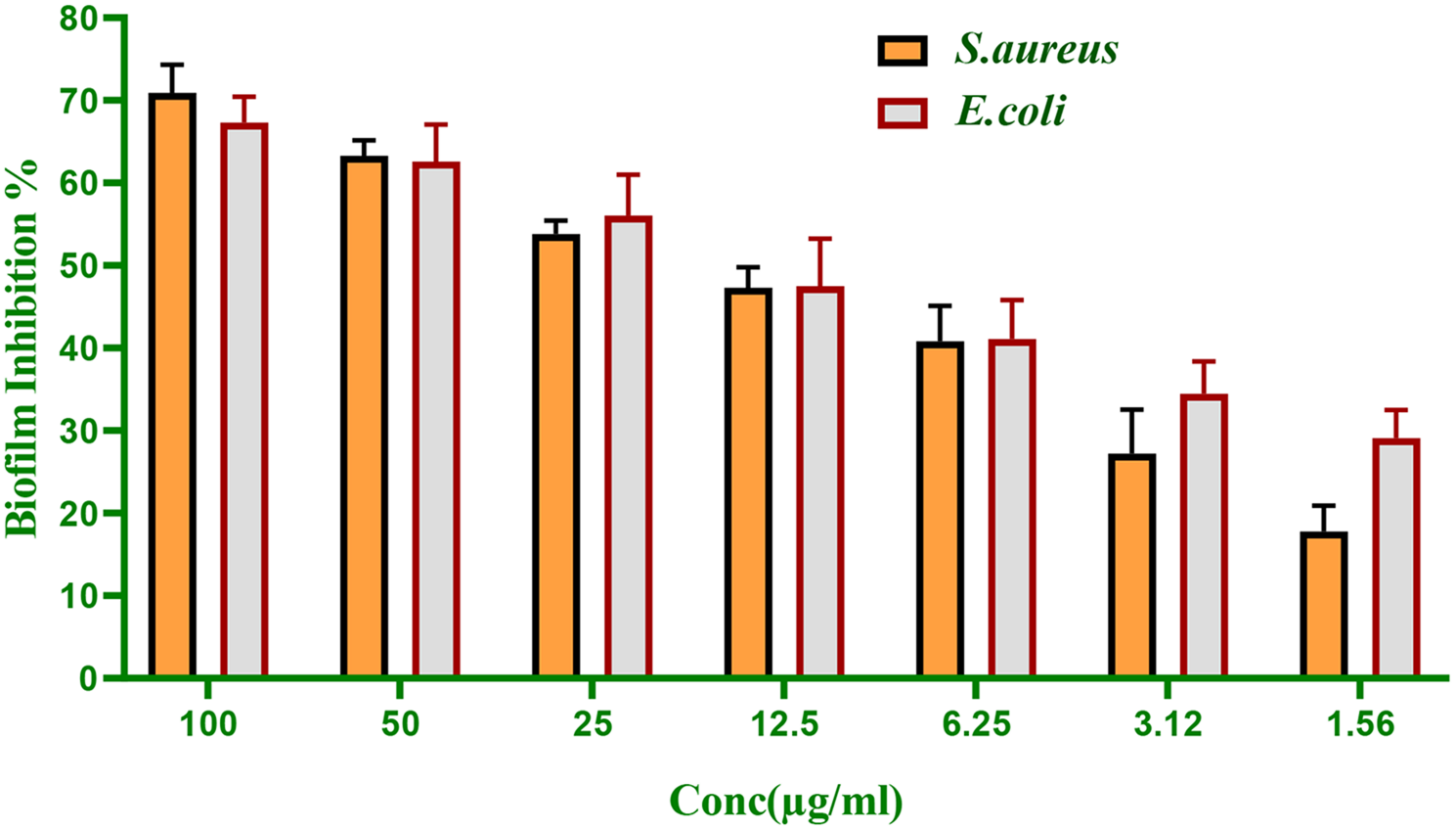
Antibiofilm assay of CuO NPs versus biofilm forming strains

**Fig. 6:**
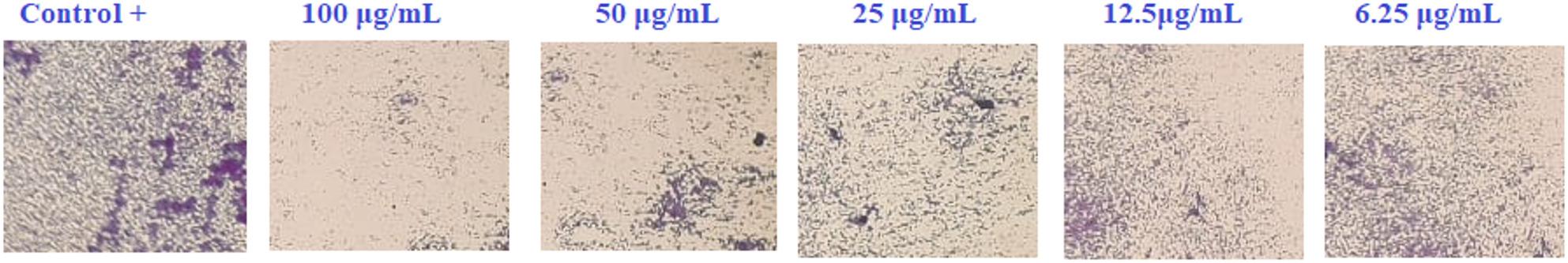
Images via inverted microscopic for the biofilm damage of S. aureus at numerous concentrations

A major contributor to antibiotic resistance in pathogenic bacteria is their ability to form biofilms. To address illnesses caused by biofilm-forming bacteria, the breakdown or elimination of biofilms is vital, since these microorganisms exhibit resistance to traditional-antibiotics, necessitating the pursuit of new-biofilm objectives [99]. The influence of CuO NPs upon biofilm development was assessed by cultivating-biofilms with varying concentrations of NPs, thereafter, staining the adherent cells with crystal-violet. Figures 5 and 6 demonstrate that increasing the concentration of nanoparticles markedly inhibited biofilm formation in the target bacteria. At 100 µg/mL, biofilm development was reduced by 70.93% in *S. aureus* and 67.3% in *E. coli*. The therapeutic value of CuO NPs as biofilm offenders has been demonstrated in a variety of investigations [100]. The CuO NPs exhibited biofilm reduction levels of 96%, 90%, 89.60%, along with 72.10% towards *Micrococcus luteus*, *Bacillus halodurans*, MRSA, as well as *E.coli*, correspondingly, at a dose of 3 mg/mL [101]. In another study demonstrated that CuO NPs exhibit remarkable anti-biofilm behavior by reducing the development of biofilm by 49% and 59% toward *K oxytoca* and *E coli*, accordingly. In addition to, the CuO NPs shown activity versus biofim of *Bacillus cereus* along with *Staphylococcus aureus* [100]. Chaieb et al. lately found that the suppression of virulence factors was more significant in the investigation of biofilm suppression. The suppression of quorum sensing, polysaccharides, enzymes, exopolysaccharides, and additional factors associated with virulence was achieved by CuO NPs [102]. Kaweeteerawat et al. assessed that the system by which metal oxide nanoparticles prevent biofilms may result from their interaction with bacterial cell membranes, hence inducing oxidative stress [103]. Prior work elucidated the anti-biofilm action by SEM pictures that exhibited deformation, increased exterior cell roughness, and shrinking of bacterial cell walls. Furthermore, biofilm formation and the number of viable cells were decreased [104].

### Antioxidant efficacy of CuO NPs

**Fig. 7:**
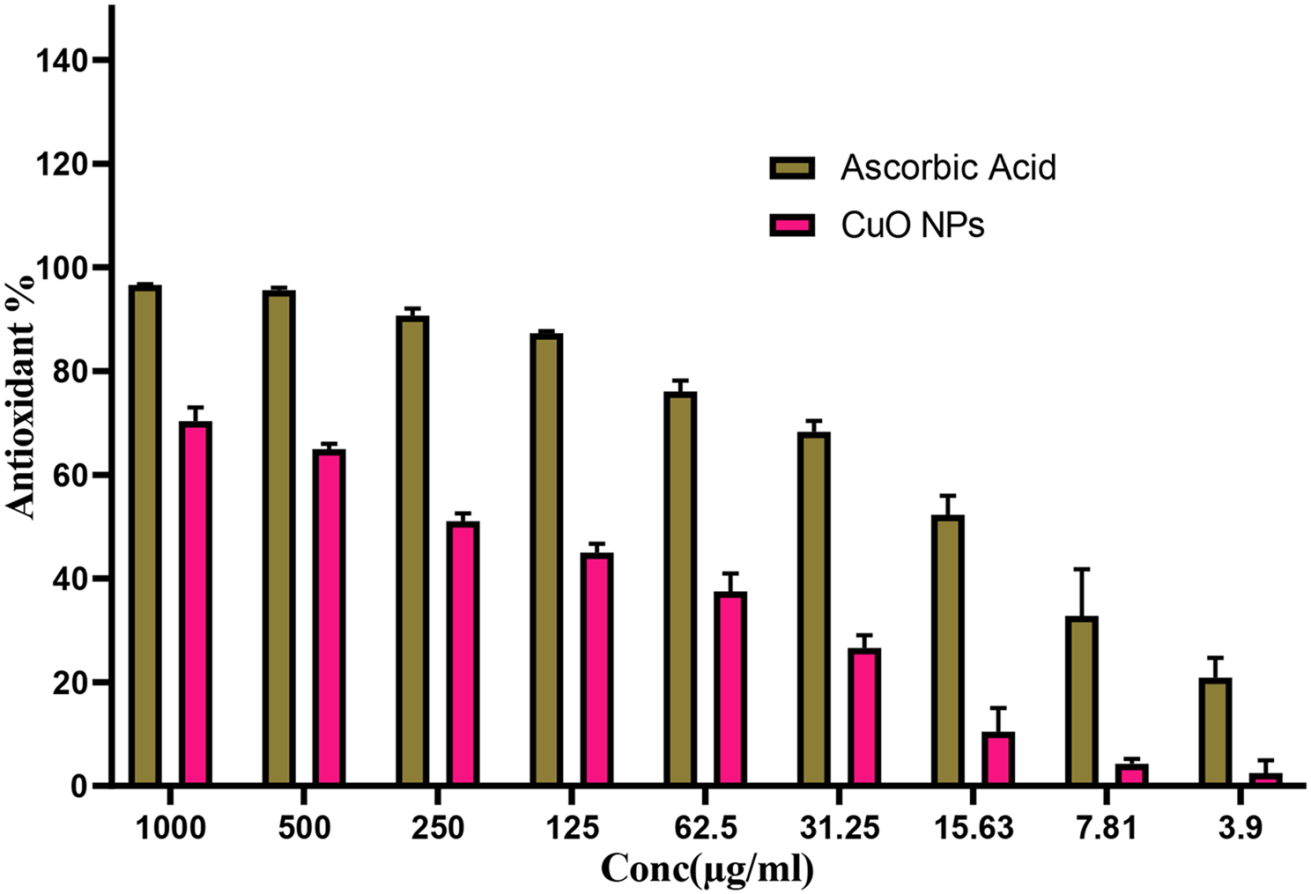
Antioxidant assay of CuO NPs via DPPH

The capacity of CuO NPs as antioxidant agent is employed by employing the DPPH radical technique. Figure 7 illustrates that CuO NPs had considerable inhibitory effect against DPPH, with an value of IC_50_ at 244.5 µg/mL. The findings were contrasted with ascorbic acid, a typical antioxidant having an IC_50_ value of 14.2 µg/mL. Consistent with our results, CuO NPs biosynthesized using *Sargassum longifolium* exhibited an inhibitory proportion of 20% whenever evaluated at a concentration of 5 g/mL [105]. Conversely, the antioxidant properties of copper mixture oxide (CuO/Cu2O) nanoparticles generated by *Phoenix dactylifera* plants exhibited significant DPPH suppression at 4 mM, with value of IC_50_ about 492 µg/mL [106]. Antioxidants substances have been used to alleviate the detrimental behavior of ROS-induced inflammation, particularly to diminish alterations and destruction inflicted on host tissue [107, 108]. Consequently, the strongest antioxidant efficiency was seen as the greatest dosage of the utilized antioxidants. Nevertheless, conventional and plant extracts exhibited a markedly elevated proportion of scavenging action. There are comparable results that was had the same trend of ability of nanomaterials of CuO to be an antioxidant substances [109]. Ascorbic acid is a recognized antioxidant, while *Camellia sinensis* as well as *Prunus africana* possess polyphenols and other phytochemicals with significant antioxidant properties, used for their natural antioxidants for the management of degenerative disorders [110]. Therefore, it is essential that our investigation demonstrates the synthesized nanoparticles’ capacity to mitigate the DPPH radical and their involvement in reducing the survival of bacteria. This requires further examination, since antioxidants are recognized as defenders of the host organism toward sicknesses. Furthermore, DPPH scavenging assays revealed that the green-synthesized CuO NPs possessed potent antioxidant properties. When tested at 40 µg mL − 1, the nanoparticles inhibited 83.9% of radicals, a performance approaching that of the reference antioxidant, ascorbic acid (95.2% at the same dose) [97].

### Antidiabetic assay

**Fig. 8:**
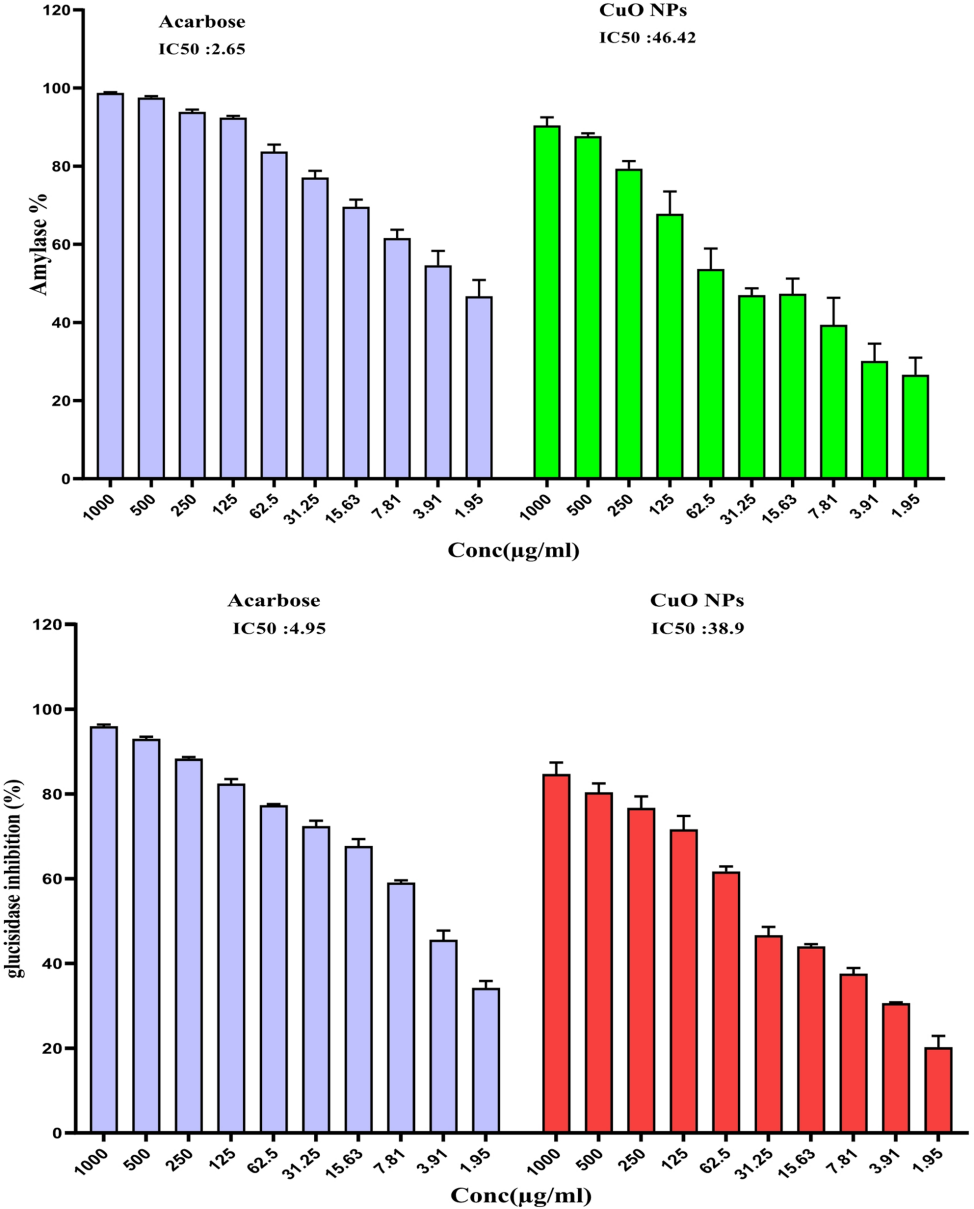
Antidiabetic activity of CuO NPs among α-amylase and α-glucosidase enzyme

Both amylase and glucosidase decompose carbs through disaccharides as well as oligosaccharides inside the gastrointestinal tract. The outcome results in increased blood glucose levels, leading to hyperglycemia among diabetic individuals. Furthermore, hyperglycemia raises the glucose generation from the liver to accommodate during metabolic activities. Still, lack of insulin action increases the glucose focus [111]. The main medications for diabetes reduce those enzymes to help to control hyperglycemia. referred to affect insulin synthesis and responsiveness as well as lower gastrointestinal tract glucose digestion include minerals (copper and zinc) and vitamins (vitamins C and D) [112, 113]. Additionally, the synthesized CuO NPs exhibited enzymes inhibiting qualities that reduced glucose absorption. subsequently was shown to suppress the activities of α-amylase and α-glucosidase by up to 90% and 84% at the maximum concentration (1000 µg/mL), comparable to the adverse effects of the widely used diabetic medication metformin. The IC_50_ values were 46.42 µg/mL for α-amylase and 38.9 µg/mL for α-glucosidase, respectively (Fig. 8). This outcome resembles that of *Cocculus hiesutus*-mediated CuO NPs [111]. In comparison to the fungal-mediated CuO NPs, it exhibited superior enzyme inhibitory efficacy [114].

### Antiviral efficacy of bio fabricated CuO NPs

**Fig. 9:**
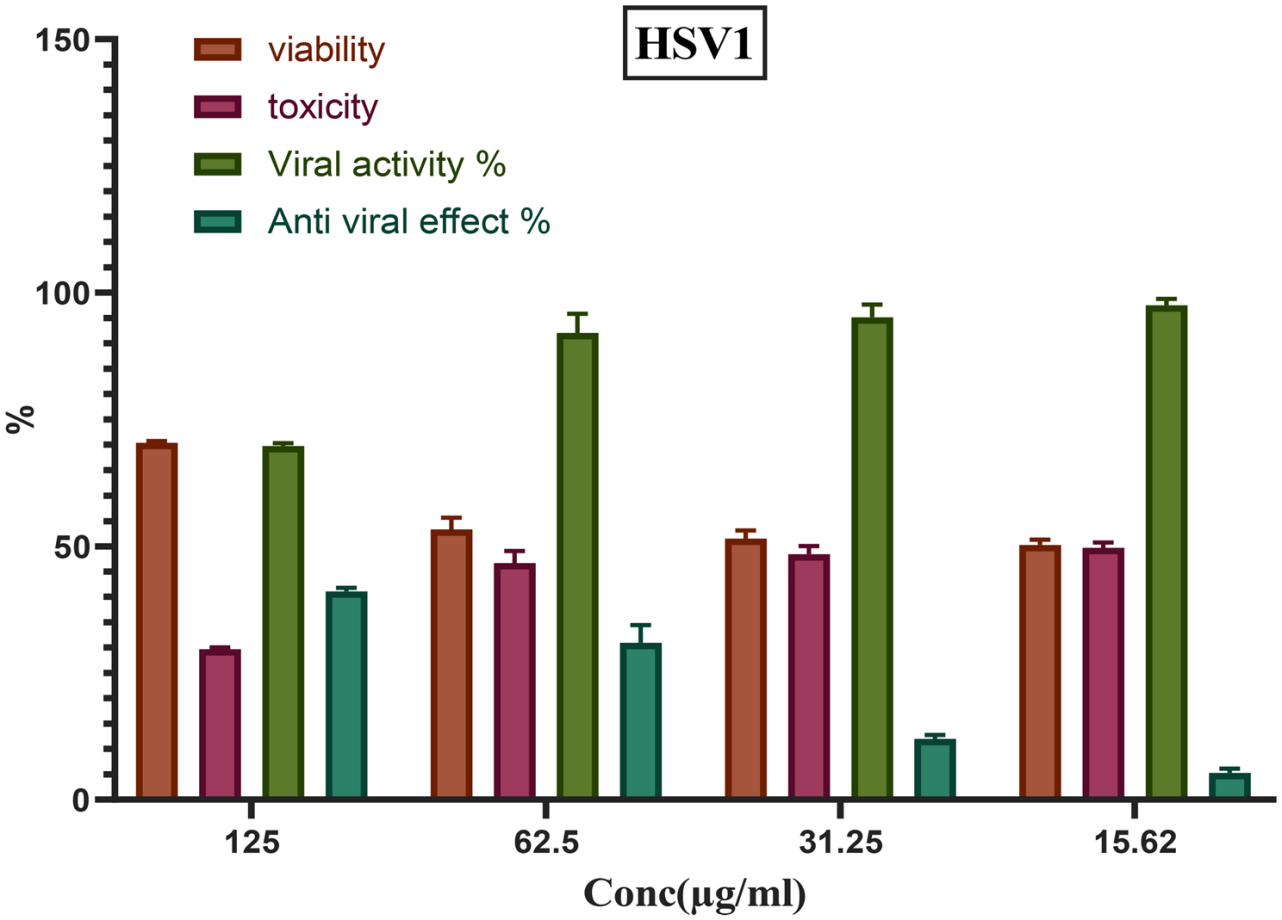
Antiviral efficacy of biofabricated CuO NPs to word HSV1

**Fig. 10:**
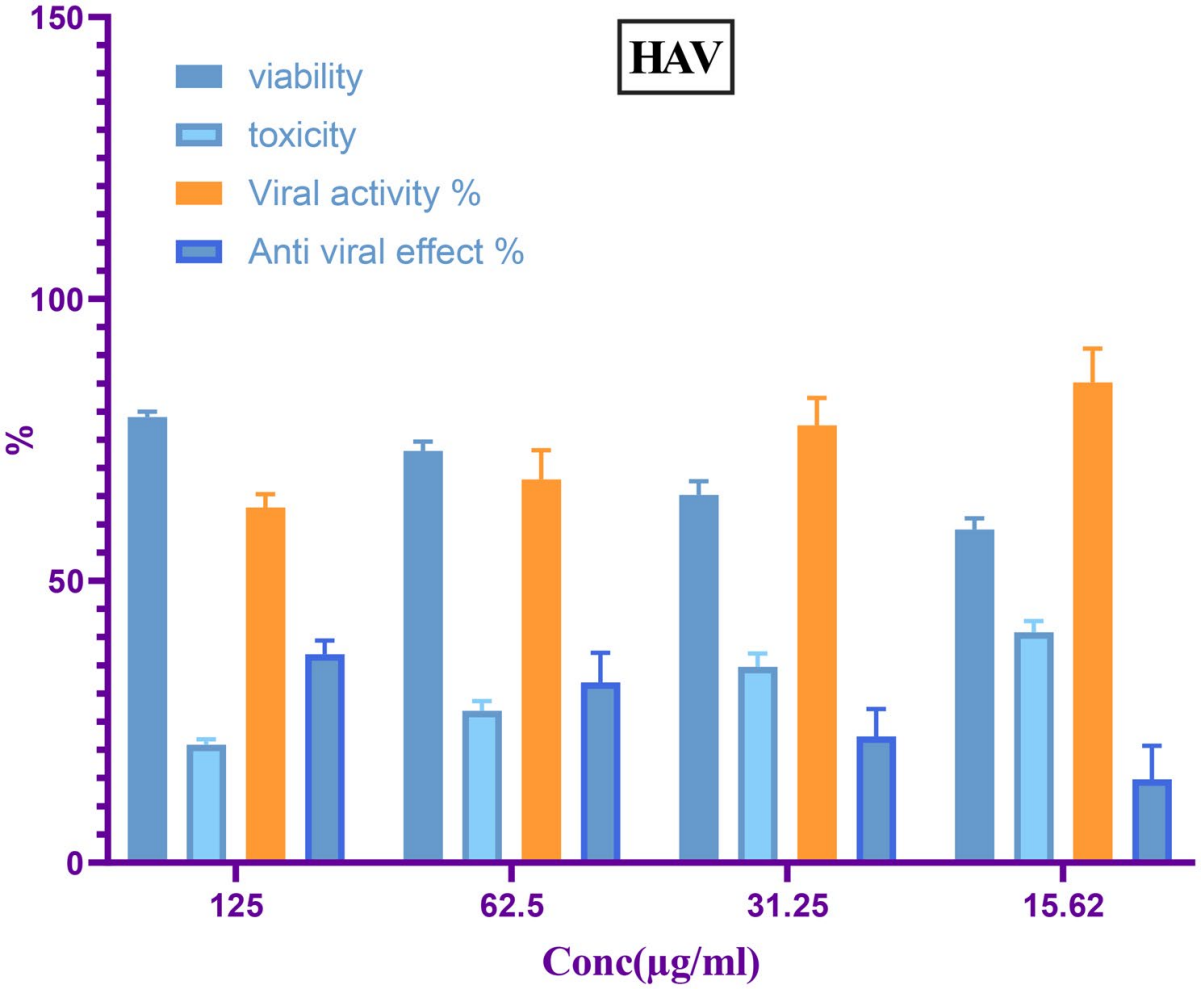
Antiviral efficacy of biofabricated CuO NPs to word HAV

Following virus adsorption, different amounts of the antiviral capacity of CuO NPs were evaluated. The research focused on a variety of doses preparations for culture cells. The suppression levels of nanomaterials of CuO among HSV1 and HAV viruses on Vero cell cultures are shown in (Figs. 9 and 10). Both kinds of examined viruses exhibited prevention using CuO NPs, but with varying degrees of viral reproduction reduction. At a quantity of 125 µg/mL, tested sample of CuO NPs had a significant suppression efficacy against the viruses, with 41.1% inhibition for HSV1 and 37% for HAV. The results indicated that CuO NPs exhibited a much higher antiviral efficacy against HSV1 compared to HAV, with varying decrease rates. The viral infection process is complex, including the virus’s attachment to its host cell to bind to cell surface receptors [115]. The mechanism of virus penetration to host cells via merging the bilayer of lipids in its cortex alongside the host cell’s external membrane. The core fusion is overseen by gB, gH, and gL [116]. Mahboub et al. reported that CNPs safeguarded Vero cells to prevent the cytopathic effects of adeno-40 and coxsackie B4 viruses, demonstrating antiviral action and inhibiting viral infection [117]. Research indicates that smaller nanoparticles have significant antiviral properties, but bigger nanoparticles demonstrate no antiviral activity [118]. Another study revealed the usefulness of environmentally friendly nanotechnology possibilities using both Ag-NPs and CuO NPs as decreasing and blocking factors for AdV-7 proliferation [119]. Additionally, it was reported the antiviral efficacy of AgNPs towards virus infection of HSV-1 and the findings indicated that the nanomaterials of silver had antiviral capability [120]. Subsequent research established the antiviral abilities of CuINPs against the *Feline calicivirus* (FCV), utilized as a surrogate for human norovirus, in *Crandell-Rees feline* kidney (CRFK) cells. Their studies have shown that CuINPs significantly diminished the vulnerability of FCV to CRFK cells. They suggested that CuINPs facilitate the production of reactive oxygen species (ROS), leading to the degradation of viral envelope proteins [121]. At the same approach in previous stud examined the blocking efficacy of CuNPs in attack to HSV-1. Their findings indicated that CuNPs suppressed HSV-1 infectiousness within a dosage-dependent way. They proposed many pathways to elucidate the anti-HSV-1 properties of CuNPs, including interactions with virus proteins and the destruction of the viral DNA [122]. The antiviral activity of CuINPs versus a H1N1 virus was studied and the virus influenced by dose antiviral efficacy of CuINPs on viral load. The work indicates that CuINPs deactivate H1N1 via interactions with viral proteins, namely hemagglutinin along with neuraminidase [123].

### Cytotoxicity and anticancer efficacy of biofabricated CuO NPs

**Fig. 11:**
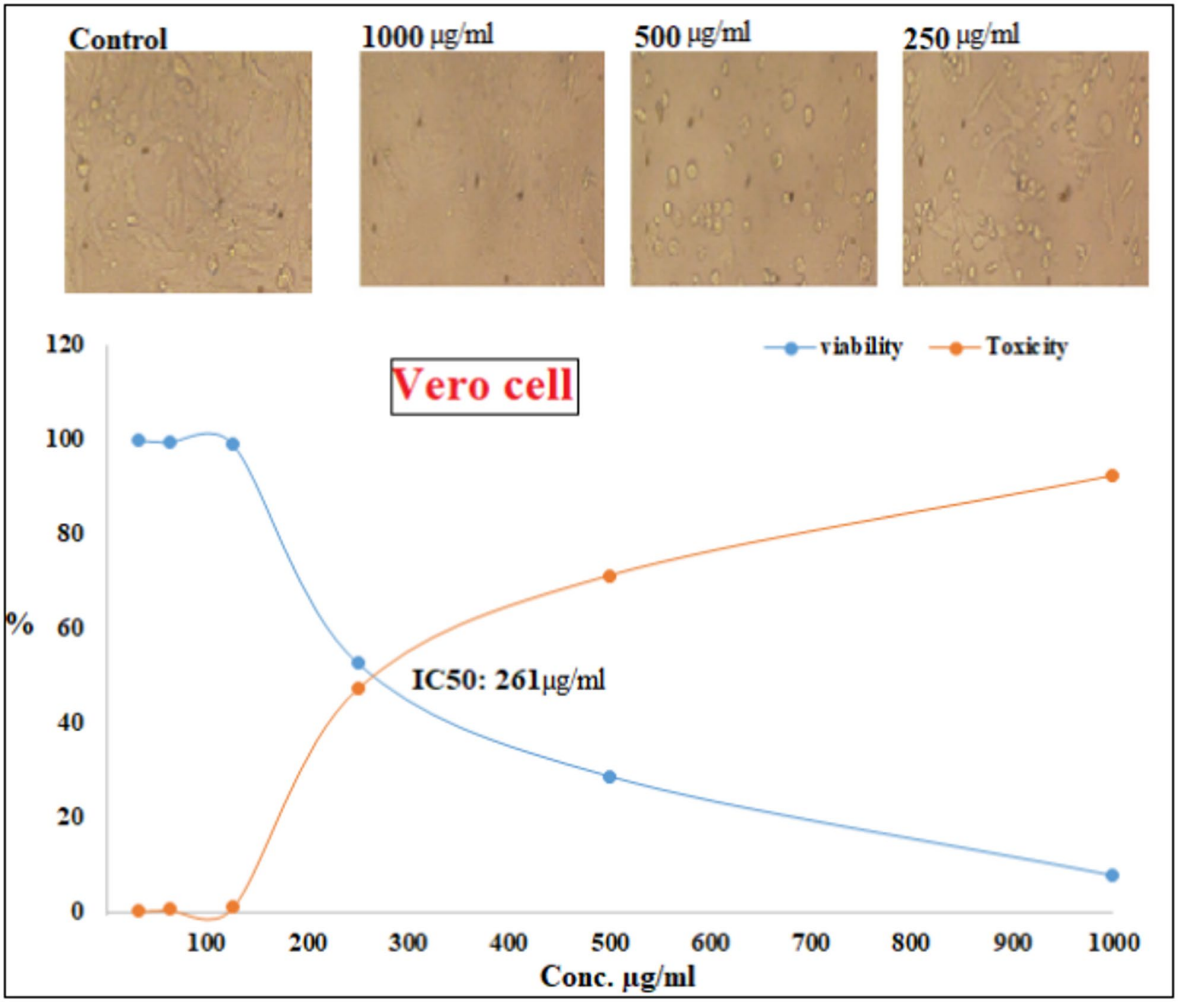
Cytotoxicity of bio fabricated CuO NPs

Vero cell lines were used to investigate the cytotoxic effects of the CuO NPs. The findings demonstrated a dependent on cytotoxic dosage of CuO-NPs, suggesting that as the quantity of tested sample of CuO-NPs increases, the percentage of normal cells falls. The outcomes indicated that at 125 µg/mL, the Vero cell lines exhibited less than 1% viability as a hazardous substance, and the IC_50_ was 261 µg/mL Fig. 11. The increasing popularity of metallic nanoparticles in biological processes has heightened concerns over their related nanotoxicity.

**Fig. 12:**
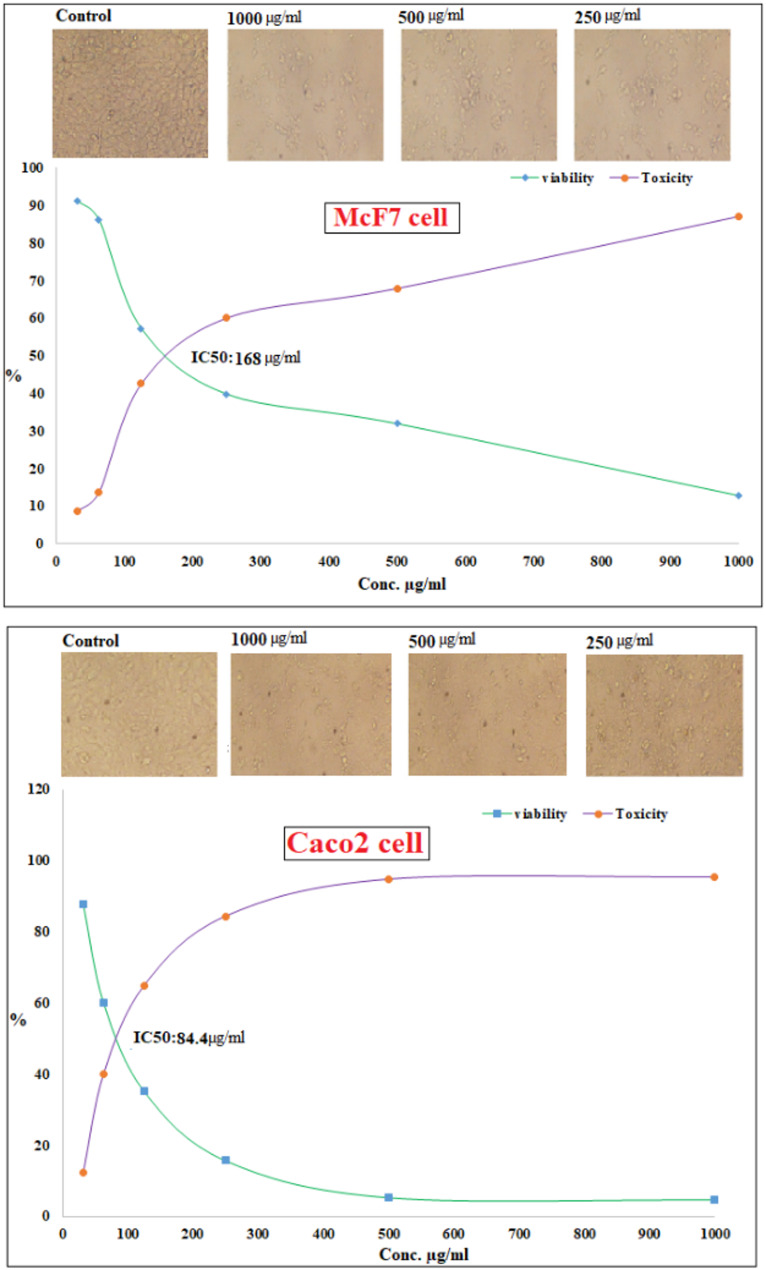
Anticancer efficacy of bio fabricated CuO NPs against McF7 and Caco2 cancer cells

The evaluation of the anticancer efficacy of CuO NPs was conducted by subjecting breast cancer cells (MCF7) and colon cancer cells (Caco-2) to varying dosages of CuO NPs, with assessment performed using the MTT test. The outcomes findings of the anticancer efficacy shown in Fig. 12. Nanoparticles of CuO were elevated cytotoxicity effect among Mcf7 and Caco2 cells at significant test doses. The cytotoxicity of CuO NPs escalated in a manner that was dependent on both time and concentration. Moreover, the value of IC_50_ was exhibited at 168 and 84.4 µg/mL for Mcf7 and Caco2 cells, respectively. Excessive creation and prolonged consumption of nanoparticles pose significant threats to human wellness [124]. A comparable sequence of toxicity was noted in the prior report; nevertheless the percentage of viable cells was elevated in that study [125]. The diminished cell viability in the current investigation may be attributed to the smaller size of the synthesized CuO NPs, facilitating their passage into cells and consequently leading to an increased rate of cell death. In the event of increased nanoparticle infiltration into cells, enzymes are released from the cell due to compromised membrane integrity, resulting in cell death. When cells are exposed to increasing concentrations of CuO NPs, the levels of reactive oxygen species (ROS) rise, leading to heightened oxidative stress. Oxidative stress mostly originates in the mitochondria, resulting in the onset of apoptosis inside the cell [125]. The CuO NPs have a superior affinity for cancer cells, resulting in enhanced cellular uptake compared to their microparticle counterparts.

A prior work shown that surface-functionalized CuO NPs significantly disrupted the cell cycle inside the MCF-7 cancer cell line within in vitro circumstances [126]. CuO NPs, capped with biomolecules from *Calotropis sinensis* as well as *C. procera*, exhibited significant cytotoxicity and caused apoptosis in several human cell lines, which involves kidney fibroblast cells (BHK21), mammary adenocarcinoma (MCF7), adenocarcinoma alveolar basal epithelial (A549), alongside adenocarcinoma (HeLa) cell lines [127, 128]. Moreover, CuO NPs with a size under 100 nm may infiltrate and collect in cellular organelles such as vesicles along with the nucleus, as well as disperse throughout the cytoplasm of human cells [129]. In the same way, it was revealed that cancer fighting experiments conducted on MCF-7 as well as normal NIH/3T3 cells revealed CuO–GO’s enhanced cytotoxicity towards cancer cells, while exhibiting negligible effects on normal cells, indicating preferential cytotoxicity [130]. Furthermore, green-synthesized CuO NPs show promise for effective breast cancer treatment, using their distinctive characteristics and eco-friendly synthesis techniques for improved applications in medicine [131].

## Conclusion

The present research examined the properties of phytosynthesis CuO NPs using *Z. Officinale* extract and their applicability. *Z. officinale* extract phytochemical and GC-mass analysis were reported. CuO confirmed a nanomaterial via characterization using various methods. CuONPs showed biofilm suppression toward two biofilm producing stains, these nanoparticles were also effective against *P. aeruginosa*, *E. coli*,* K. pneumoniae*,* E. faecalis*,* B. subtilis*, and *S. aureus*. Their antioxidant capabilities and free radical scavenging activity are evident, with an IC_50_ of 244.5 µg/mL. CuO NPs demonstrated higher anti-diabetic efficacy via suppression assays of antidaibetc enzymes. Additionally, CuO NPs showed low toxicity on Vero cells and possible anticancer effect against CaCo2 and McF7 carcinoma cell lines. CuO NPs at 125 µg/mL showed strong antiviral activity toward HAV and HSV1, with 37% and 41.1% activity, respectively, suggesting their potential in biological applications.

## Data Availability

The data used to support the findings of this study are available from the corresponding author upon request.
